# Cross-sectional associations between accelerometry-measured physical activity and physical fitness among elementary school children

**DOI:** 10.3389/fspor.2026.1761419

**Published:** 2026-07-03

**Authors:** Karin Helene Danielsen, Kim Arne Heitmann, Edvard H. Sagelv, Thilde Kleppe Vårnes

**Affiliations:** 1 Department of Education, UiT The Arctic University of Norway, Tromsø, Norway; 2 School of Sport Sciences, UiT The Arctic University of Norway, Tromsø, Norway; 3 Western Norway University of Applied Sciences, Bergen, Norway

**Keywords:** accelerometer, actigraph, schoolchildren, test of physical fitness, weight status

## Abstract

Physical activity (PA) is essential for children's physical and mental health, with moderate-to-vigorous physical activity (MVPA) playing a key role in improving physical fitness (PF). Despite its benefits, PA levels among children are insufficient, and PF levels have declined, highlighting the need for targeted interventions. PA was measured using accelerometers and expressed as total PA (counts per minute (cpm) and MVPA (min·day^−1^). PF was measured using the Test of Physical Fitness (TPF). The primary objective was to examine (a) the association between device-measured PA and PF in school-aged children (6–12 years), b) to explore the extent to which this association can inform the amount of PA needed to influence TPF scores, and (c) whether sex, age, or body mass index (BMI) modifies the association between PA and PF. A sample of 216 school children aged 6, 8, 10 and 12 years from Northern Norway participated. Multivariable linear regression analyses were conducted, adjusting for sex, age, BMI, wear time, and school. In multiple adjusted analyses, we observed an association between children's PA level and TPF. MVPA was positively associated with PF in the overall population and across sex, age, and BMI subgroups, whereas the association of total PA with PF was significant only for boys and girls, older children, and normal weight children. These findings emphasize the importance of promoting PA, especially MVPA, to enhance PF in children, with tailored approaches for younger, overweight, and less active children.

## Introduction

Physical activity (PA) in children is closely related to physical fitness (PF), including cardiorespiratory fitness, musculoskeletal fitness, motor skills, and, in some cases, also body composition (1, 2). Both PA and PF play an important role in children's physical and psycho-social growth and development (3–5), contributing to an enhanced health-related quality of life (6, 7). This early stage of childhood is critical for establishing both physical and mental health, as it is characterized by significant changes. Inadequate PF can affect children and, in some cases, negatively impact their academic achievement (8–10). Engaging in PA during these formative years can positively impact cardiometabolic risks and other health indicators, potentially carrying these benefits into adulthood (11, 12).

However, current research indicates that PA levels among children are generally low, and children spend much of their waking hours being sedentary (13–15). Only 29% of European children and adolescents adhere to the global PA guidelines, which recommend 60 min of moderate-to-vigorous physical activity (MVPA) each day (15), and PA levels have been observed to decline with increasing age (16, 17). Furthermore, prior research highlights notable sex differences in PA, with boys being more physically active compared to girls (15, 18–22). Daily PA has been linked to aerobic capacity in boys, whereas the relationship appears to be unclear or absent in girls (21). Furthermore, a higher body mass index (BMI) has been associated with reductions in both cardiorespiratory fitness and overall (PA) levels (22–25), and intermittent, accumulated moderate-intensity PA may play a more significant role than sustained vigorous-intensity PA in influencing children's fitness and fatness levels (22). Higher levels of PA are generally associated with better performance, while fat mass stands out as the most significant negative predictor of children's PF. In contrast, fat-free mass and a 10-min increase in daily moderate-to-vigorous physical activity (MVPA) has been shown to correspond to 2–6 laps of improved performance (25).

Assessing PF in children provides critical insights for developing and implementing clinical and public health strategies to promote and maintain their well-being. While laboratory tests are considered the gold standard for evaluating aerobic capacity, strength, power, coordination, and agility, their high costs, time demands, and simulated settings limit widespread application (26, 27). The Test of Physical Fitness (TPF) provides valid and reliable measures of children's fitness level (28). This is a nine-item test battery that aims to provide reliable, objective measures of children's everyday activities such as jumping, throwing, climbing, and running.

Despite the known link between PA and PF in children, only one study has investigated the association between PA in conjunction with the TPF. Vainauskas (29) observed that higher PA level among children was associated with better results in two of the TPF test items (tennis ball throw and the 10 × 5 m shuttle run test). However, children's PA level was assessed by a parental questionnaire. To our knowledge, no studies have examined objectively measured PA and TPF.

This study aims to examine a) the cross-sectional association between objectively measured PA and TPF in Norwegian schoolchildren, b) to explore the extent to which this association can inform the amount of PA needed to influence TPF scores, and c) whether sex, age, or BMI modifies the association between PA and PF.

## Materials and methods

### Study design

This cross-sectional study included children recruited from two elementary schools in Tromsø municipality, Northern Norway. To ensure a representative spread among the schoolchildren, we invited all children (*n* = 283) aged ∼6 years (1st grade, *n* = 57), ∼8years (3rd grade, *n* = 50), ∼10 years (5th grade, *n* = 55), and ∼12 years old (7th grade, *n* = 54) to participate. 216 school children provided valid accelerometry data, completed all items in TPF, and underwent anthropometric measurements.

All the measurements were conducted by class, with the class teacher present, and were carried out in the school's sports hall. Before the physical tests, the children's height and weight were measured, and an accelerometer was attached. The test execution took approximately an hour and was conducted with the children rotating between various stations led by researchers, physical education students, and schoolteachers from the local school. In advance, all observers underwent theoretical and practical training to secure the validity and reliability of the observers.

### Physical fitness

Physical fitness was measured using the TPF (28). The TPF is a nine-item test battery assessing key areas of children's PF, including strength, endurance, motor coordination, balance, and agility, engaging both the upper and lower body to gain a holistic understanding of the children's functional capacity (28). A description of the nine-item test battery is presented in Table 1.

**Table 1:** Description of the nine test items in the test of physical fitness (TPF).

Test item	A measure for	Test description
Standing broad jump (cm)	Explosive power	The child stands with his/her feet parallel and jumps with both feet simultaneously as far forward as possible. The test item score (best of 2 attempts) is the distance between the starting line and the landing position.
Jumping on 2 feet (s)	Leg muscle power	Jumping a distance of 7 m on 2 feet as fast as possible. The test score (best of 2 attempts) is the time needed.
Jumping on 1 foot (s)	Leg muscle power	Jumping a distance of 7 m on one foot (the child is free to choose which foot) as fast as possible. The test score (best of 2 attempts) is the time needed.
Throwing a tennis ball (m)	Arm muscle power	Throwing a tennis ball with one hand (the child chooses which hand) as far as possible. The test item score (best of 2 attempts) is the distance thrown.
Pushing a medicine ball (m)	Upper body strength	Pushing a medicine ball (1 kg) with two hands as far as possible from the chest. The test item score (best of 2 attempts) is the distance achieved.
Climbing wall bars (s)	Coordination	Climbing up the wall bar, crossing over two columns to the right, and climbing down the fourth column as fast as possible. The test score (best of 2 attempts) is the time needed.
Shuttle run (s)	Agility	Run 10 × 5 m. The test item score (best of 2 attempts) is the time required to run the distance.
Running 20 m (s)	Speed	Running 20 m as fast as possible. The test item score is the time required to run the distance.
Reduced Cooper test (m)	Endurance	The child runs or walks around the marked rectangle measuring 9 × 18 m (the size of a volleyball field) for 6 min. Both running and walking are allowed. The test item score is the distance covered in 6 min.

To present the overall fitness performance in one score, the performance of each test item was transformed into a standardized z-score based on the sample mean. Thereafter, we summarized the nine z-scores and calculated the average z-score performed by each child across all nine items. The average z-score is presented as the total TPF score. A higher total TPF score indicates better overall performance (28). The TPF has previously been validated. Test-retest correlation of total TPF score was 0.90 (*p* < 0.001), and the construct validity of the test was *r_s_* 0.93 for girls and 0.89 for boys (*p* < .0001 for both) (28). We did not examine inter-rater reliability of the observers who performed the TPF assessment in this current study.

### Physical activity

Physical activity was measured using a triaxial ActiGraph wGT3X-BT accelerometer (ActiGraph LLC, Pensacola, Florida, USA; sampling frequency: 30 Hz). The device has demonstrated validity for measuring PA in children (30). The accelerometer was initialized, and data was downloaded using the ActiLife v.6.13.3 software program (ActiGraph, LLC, Pensacola, Florida, USA). The accelerometer was fitted to each child with a band around their right hip. The children were instructed to wear the monitor for all waking hours over seven consecutive days (five weekdays and two weekend days), except during water-related activities (e.g., swimming, showering etc.).

The devices were set to start recording at 06:00 on the day after the monitors were handed out to prevent potential accelerometry reactivity (abnormally high activity) during the first hours after receiving the accelerometer (31). All activity data between 00:00 and 06:00 was excluded. The raw acceleration was reduced to 10-second epoch intervals. Blocks of ≥20 min without any registered activity [0 counts per minute (cpm)] were considered as non-wear time and excluded from further analyses (32), and these were excluded manually in Excel spreadsheets (Microsoft Corporation, Redmond, WA, USA). Inclusion criteria for data analysis were ≥2 days wear time with ≥8 h of recorded data each day (15). Total PA was expressed as cpm and included all accelerations to which the monitor had been exposed, divided by valid wear days. We used the threshold of >2,000 cpm to define MVPA, which has been validated for children aged 5–15 years and demonstrates acceptable classification accuracy (32, 33). This threshold is approximately equivalent to a walking pace of 4 km/h in youth (33, 34), and have been used in previous studies (15, 16, 35, 36).

### Anthropometric measurements

The children wore light clothing and no shoes during the anthropometric measurements. Weight was measured to the nearest 0.1 kg (Seca 877, Seca GmbH & Co. KG, Germany) and height to the nearest 0.5 cm (Seca 213, Seca GmbH & Co. KG, Germany). BMI was calculated as weight divided by height squared (kg/m^2^) and further divided into normal weight (isoBMI < 25 kg/m^2^) and overweight and obese (isoBMI ≥ 25 kg/m^2^) according to age and sex (37).

### Statistical analysis

Descriptive characteristics are presented as standard deviations (SD). The association between PA (total PA per 50 cpm·day^−1^ increase) and MVPA (10 min·day^−1^ increase) and TPF z-score was estimated by univariable and multivariable linear regression analyses. The associations are presented as standardized beta-coefficients (b) with 95% confidence intervals (CI). We analyzed two different models: Model 1 was unadjusted, and Model 2 was adjusted for sex, age, BMI, accelerometer wear time, and school.

We tested for possible interactions between “PA×sex”, “PA × age group” (6–8 years and 10–12 years), and “PA × normal and overweight”, in the association between PA (total PA or MVPA) and TPF score, with no significant interactions (*p* > 0.211). However, to present sex-,age-, and weight-specific associations between PA and PF, we stratified our main analyses by these variables.

Significance level was set to *p* < 0.05. Statistical analyses were performed using the IBM Statistical Package for Social Sciences, version 29 (IBM SPSS Statistics, Armonk, NY, USA).

### Ethical considerations

Participation was voluntary for the schoolchildren, and the children had the opportunity to withdraw from all measurements if they did not want to participate. Written information about the study was sent in advance to the parents through the schoolteachers, and participation required written approval from the parents/legal guardians. The Norwegian Agency for Shared Services in Education and Research approved the storage and handling of personal data (reference number: 56212), and the study was carried out in accordance with the Declaration of Helsinki. As the sample in our study included young children, we chose not to include any questions about ethnicity or socioeconomic background, as these could be difficult to answer or be perceived as discriminatory by the children. We were also aware that objective measures of PF could create room for comparison between schoolchildren, even though we did not state the results. Ethical considerations were assessed in relation to the measurement of weight and height. To avoid comparisons, the schoolchildren were measured individually in a separate room.

## Results

Descriptive statistics for all participants are presented in Table 2. A total of 216 children aged 6, 8, 10 and 12 provided valid data on accelerometry data and TPF score, which are included in our analysis.

**Table 2:** Characteristics of participants stratified by weight, sex, and grade.

Variables	Total	Girls	Boys	1st–3rd grade	5th–7th grade	Normal weight	Overweight
	(*n* = 216)	(*n* = 109)	(*n* = 107)	(*n* = 107)	(*n* = 109)	(*n* = 168)	(*n* = 48)
Age (y)	9.43 (2.26)	9.33 (2.29)	9.41 (2.32)	7.30 (1.03)	11.43 (1.11)	9.30 (2.34)	9.53 (2.19)
Height ((cm)	136.72 (14.02)	136.21 (14.05)	137.26 (14.03)	124.92 (7.03)	148.33 (8.34)	135.79 (13.88)	140.01 (14.32)
Weight (kg)	34.48 (11.66)	34.55 (11.79)	34.39 (11.57)	25.77 (5.42)	43.01 (10.00)	31.67 (9.04)	44.39 (14.40)
BMI (kg/m^2^)	17.92 (3.19)	18.09 (3.20)	17.74 (3.19)	16.43 (2.04)	19.42 (3.39)	16.84 (1.67)	22.01 (3.79)
MVPA (min-day^−1^)	58.38 (24.07)	53.00 (20.79)	63.92 (25.98)	61.64 (23.89)	55.53 (24.01)	60.37 (24.04)	52.19 (23.24)
Total PA (cpm)	620.69 (155.17)	590.55 (128.71)	651.69 (173.47)	674.99 (133.43)	568.33 (158.29)	631.55 (153.39)	587.07 (159.42)
TPF (mean z-score)	0.01 (0.88)	−0.18 (0.88)	0.21 (0.87)	−0.60 (0.51)	0.62 (0.66)	0.07 (0.90)	−0.19 (0.74)
Wear Time (min-day^−1^)	593.03 (103.44)	596.00 (95.34)	590.97 (111.20)	592.23 (99.36)	593.98 (107.32)	597.04 (102.23)	580.13 (107.20)

Data are shown as mean ± standard deviation. BMI, Body Mass Index; MVPA, moderate-to-vigorous physical activity; Total PA (cpm), Total physical activity counts per minute; TPF, Test of Physical Fitness mean z-score.

### Associations between total PA, MVPA, and TPF in the whole group

In unadjusted analyses, there were no significant associations between neither total PA (50 cpm·day^−1^) or MVPA (10 min·day^−1^) and TPF score (Table 3).

**Table 3:** Associations between PA and TPF score.

Group variables	Model 1 unadjusted			Model 2 adjusted			Effect size
	b (95% CI)	*P*	*R* ^2^	b (95% CI)	*P*	*R* ^2^	
Overall (*n* = 216)							
Total PA (50 cpm-day^−1^)	−0.02 (−0.06 to 0.01)	0.221	0.01	0.05 (0.03 to 0.06)	<0.001	0.79	Large
MVPA (10 min-day^−1^)	0.05 (0.01 to 0.10)	0.022	0.02	0.07 (0.05 to 0.10)	<0.001	0.79	Large
Boys (*n* = 107)							
Total PA (50 cpm-day^−1^)	−0.03 (−0.07 to 0.02)	0.316	0.01	0.05 (0.03 to 0.08)	<0.001	0.77	Large
MVPA (10 min-day^−1^)	0.05 (−0.02 to 0.11)	0.132	0.02	0.08 (0.04 to 0.11)	<0.001	0.78	Large
Girls (*n* = 109)							
Total PA (50 cpm-day^−1^)	−0.06 (−0.12 to −0.002)	0.043	0.04	0.03 (0.002 to 0.07)	0.036	0.78	Large
MVPA (10 min-day^−1^)	0.02 (−0.05 to 0.10)	0.540	0.004	0.07 (0.03 to 0.11)	<0.001	0.80	Large
1st–3rd grade (*n* = 107)							
Total PA (50 cpm-day^−1^)	0.04 (−0.002 to 0.07)	0.064	0.03	0.02 (−0.02 to 0.06)	0.322	0.15	Medium
MVPA (10 min-day^−1^)	0.07 (0.03 to 0.11)	<0.001	0.10	0.06 (0.02 to 0.11)	0.006	0.20	Medium
5th–7th grade (*n* = 109)							
Total PA (50 cpm-day^−1^)	0.06 (0.02 to 0.10)	0.002	0.09	0.05 (0.01 to 0.09)	0.008	0.26	Large
MVPA (10 min-day^−1^)	0.11 (0.06 to 0.16)	<0.001	0.16	0.09 (0.04 to 0.14)	<0.001	0.29	Large
Normal weight (*n* = 168)							
Total PA (50 cpm-day^−1^)	−0.02 (−0.07 to 0.02)	0.277	0.01	0.04 (0.02 to 0.06)	<0.001	0.80	Large
MVPA (10 min-day^−1^)	0.05 (−0.002 to 0.11)	0.057	0.02	0.07 (0.04 to 0.10)	<0.001	0.81	Large
Overweight (*n* = 48)							
Total PA (50 cpm-day^−1^)	−0.04 (−0.10 to 0.03)	0.234	0.03	0.03 (−0.01 to 0.08)	0.116	0.68	Large
MVPA (10 min-day^−1^)	0.03 (−0.05 to 0.12)	0.440	0.01	0.08 (0.02 to 0.13)	0.008	0.71	Large

Data are shown as standardized beta coefficients (b) with 95% CI. Model 1 is unadjusted, Model 2 is adjusted for sex, age, BMI, Body mass Index, school grade, and accelerometer wear time. Total PA (50 cpm), Total physical activity 50 count per minute; MVPA, moderate-to-vigorous physical activity 10 min per day, Effect size Value 0.01 Small effect. 0.09 Medium Effect and 0.25 Large Effect.

In the multivariable adjusted model, the children had a 0.05 (95% CI: 0.03–0.06. *p* < 0.001) higher TPF score for every 50 cpm higher total daily PA, and a 0.07 (95% CI: 0.05–0.10. *p* < 0.001) higher score for every 10 min higher daily MVPA (Table 3).

### Associations between total PA, MVPA, and TPF split by sex

When stratified by sex, only girls had a 0.06 (95% CI: −0.12 to −0.002. *p* = 0.043) reduced TPF score for every 50 cpm higher daily total PA in the unadjusted model (Table 3). While there were no significant associations between MVPA and TPF score for either boys or girls.

In the adjusted model, boys had a 0.05 (95% CI: 0.03–0.08. *p* < 0.001) higher TPF score for every 50 cpm higher daily PA, and a 0.08 (95% CI: 0.04–0.11. *p* < 0.001) for every 10 min higher daily MVPA (Table 3). Among girls, TPF score increases with 0.03 (0.002–0.07) for every 10 cpm higher daily PA, and with 0.07 (95% CI: 0.03–0.11. *p* < 0.001) for every 10 min of daily MVPA increase (Table 3).

### Associations between total PA, MVPA, and TPF split by age

When stratified by age, children aged 6–8 years (1^st^–3rd grade), only MVPA was associated with TPF score, increasing with 0.07 (95% CI: 0.03–0.11. *p* *<* 0.001) for every 10 min higher daily MVPA in the unadjusted model (Table 3). For children aged 10–12 years (5th to 7th grade), TPF score were 0.06 (95% CI: 0.02–0.10. *p* = 0.002) higher for every 50 cpm increase of total PA, and 0.11 (95% CI: 0.06–0.16. *p* < 0.001) higher TPF score for every 10 min of daily MVPA increase (Table 3).

In the adjusted model, for children aged 6–8 years (1^st^–3rd grade), there was no association between total PA and TPF score, while TPF score was 0.06 (95% CI: 0.02–0.11. *p* = 0.006) higher for every 10 min of daily MVPA increase (Table 3). Among older children aged 10–12 years (5th to 7th grade), they had a 0.05 (95% CI: 0.01–0.09. *p* = 0.008) higher TPF score for every 50 cpm higher total daily PA, and a 0.09 (95% CI: 0.04–0.14. *p* < 0.001) higher TPF score for every 10 min higher daily MVPA (Table 3).

### Associations between total PA, MVPA, and TPF split by weight

For the unadjusted model, stratified by normal and overweight, there were no significant associations between either total PA, or MVPA and TPF score in any of those groups (Table 3). In the adjusted model, the TPF score was 0.04 (95% CI: 0.02–0.06. *p* < 0.001) higher for every 50 cpm total PA, and 0.07 (95% CI: 0.04–0.10. *p* < 0.001) higher between TPF score and for every 10 min higher daily MVPA in the normal weight group (Table 3). Among overweight children, there was no significant association between total PA and TPF score, while TPF score was 0.08 (95% CI: 0.02–0.13. *p* = 0.008) higher for every 10 min of daily MVPA increase (Table 3).

## Discussion

In this cross-sectional study examining the association between PA and PF, higher PA was positively associated with a higher total score in the TPF test battery, indicating that higher PA may lead to higher PF among school-aged children. Notably, MVPA was positively associated with PF in the overall population and across all sex, age, and BMI subgroups, whereas the association of total PA with PF was significant only for sex considered separately, for older children, and for normal weight children.

Overall, in the adjusted model, for every 50 cpm higher total daily PA and 10 min higher daily MVPA, children increased TPF score by 0.05 and 0.07, respectively. This is consistent with previous studies among school-children, that also found a positive association between increased PA and PF (1, 5, 21, 25). This responsiveness could be attributed to developmental changes, such as physical maturation, or to the greater cognitive and physical demands during these grades (10, 38).

When split by sex, both total PA and MVPA were positively associated with a higher TPF scores in both groups. Previous studies, such as Kidokoro (18), suggested more nuanced sex-specific patterns. For instance, their findings indicated that higher MVPA was crucial for boys to reduce low PF, whereas vigorous PA was strongly associated with PF among girls. This highlights that while general PA benefits for both sexes, the optimal type or intensity for PA may differ to achieve specific PF outcomes. These findings are supported by a study showing that boys are more active than girls, especially in high-intensity activities (22), while another study found that daily activity levels in younger children are related to aerobic fitness in boys but is unclear among girls (21).

When stratified by lower and higher aged groups/grades, MVPA was associated with PF in both groups in the adjusted model, whereas total PA and PF were only associated in children aged 10–12 years (5^th^–7th grade). This may be due to the fact that, even at this age, differences in both cognitive and physical development are beginning to emerge (39, 40). Moreover, since only MVPA was significantly associated with PF in children aged 6–8 years (1^st^–3rd grade), this could suggest that children in this age group may be particularly responsive to the benefits of time spent in MVPA. This is aligned with findings from a study on younger children, which reported that daily activity levels were associated with aerobic capacity in boys, but not in girls. Additionally, younger children generally engaged in short bursts of PA, rather than sustained activity (22).

When split by weight, total PA was only associated with PF in normal weight children, while MVPA was associated with PF in both groups. This phenomenon suggests that overweight children might need PA at higher intensities to increase their PF. These findings are consistent with previous research indicating that overweight children exhibit a more pronounced decline in cardiorespiratory fitness and PA levels from late childhood to adolescence (23). Furthermore, a recent study did not identify an association between weight and PF in children under nine years (41), implying that such an association may emerge as a part of the normal development trajectory in children. Another study found that children's fat mass index had a negative association with PF, increased body fat is associated with decreased levels of PF, while children's fat free mass index had a positive association with fitness: higher levels of PA are associated with improved PF (25) While another found that higher frequently duration and PA were positively associated with aerobic fitness, and negatively associated with body fatness (22).

Our initial unadjusted analysis showed a weakly significant association between children's PA and PF. However, after adjusting for key covariates, including sex, age, BMI, accelerometer wear time, and school grade, a significantly stronger positive relationship appeared, indicating that increased PA is independently associated with higher TPF scores. This change in significance suggests that these demographic and anthropometric factors acted as confounders or suppressor variables in the unadjusted model, hiding the true relationship between PA and PF (42). By statistically controlling these factors, our adjusted model offers a clearer and more reliable estimate of how children's PA levels contribute to their PF. The results show a stronger, more significant relationship and a high degree of explanatory power. This finding is important because it confirms the independent role of total PA (cpm) and MVPA (min-day^−1^), even when considering other factors influencing PF, and highlights the need to encourage adequate PA in children.

### Practical implications

The findings highlight the need to prioritize and promote total PA and MVPA in children's daily routines, as it appears to provide benefits for PF. This suggests that facilitating more daily activities, both total PA levels and MVPA, will improve and enhance children's PF (5, 7, 16). For instance, incorporating an additional 10 min of daily MVPA should be feasible within the school setting, both during lessons and recess. In addition, our results show that younger children (aged 6–8 years), and overweight children would need PA of higher intensities to increase their PF. However, more research is needed to explore these associations in further detail.

### Strengths and limitations

The main strength of this study is the device measured PA levels. Self-reported PA in children relies on proxy reports from parents, which are prone to recall bias (43). Consequently, our accelerometry-measured PA may reflect the actual PA levels to a greater extent than self-reported measures (30). Another strength is the use of validated, objective, and quantifiable motor skills assessments to represent a holistic understanding of the children's PF (28, 44). Finally, the broad age range strengthens the external validity of the study results for children in primary school of all ages.

Our study has some limitations that should be addressed. First, our sample was recruited from two schools in Northern Norway, which may limit generalizability to children from other geographic regions, socioeconomic backgrounds, or ethnic groups. Future studies should include more diverse samples to examine whether these associations hold across different populations. Second, as this is a cross-sectional study, it is not possible to establish cause-and-effect relationships from the results. Therefore, we cannot determine whether PA improves PF or vice versa. Nevertheless, given that PA is a behavior and PF is a biological trait, it is more likely that a PA improves PF than the opposite. Third, accelerometry-measured PA underestimates activities such as cycling due to a lack of body movement (in this case, hip movements), as well as water-based activities, since the accelerometer is not waterproof. In our study, children were instructed to remove the accelerometer when entering contact with water. Finally, as we did not examine inter-rater reliability of the test instructors in this study, subjective differences in scores may have influenced our results.

## Conclusion

This study observed that higher levels of PA, particularly MVPA, were associated with higher PF in school children. The association between PA and PF differs by age and body weight. MVPA was positively associated with PF across all subgroups, while total PA was significant only for boys, girls, older children (aged 10–12 years), and those with normal weight. This study indicates that promoting PA, especially of moderate and high intensity, in school-aged children may positively influence all components of the children's PF level.

## Data Availability

The raw data supporting the conclusions of this article will be made available by the authors, on reasonable request.
